# Circulating CircRNAs Panel Acts as a Biomarker for the Early Diagnosis and Severity of Parkinson’s Disease

**DOI:** 10.3389/fnagi.2021.684289

**Published:** 2021-07-01

**Authors:** Lingling Zhong, KeJu Ju, Ainian Chen, Hua Cao

**Affiliations:** Department of Neurology, The Affiliated Huai’an No. 1 People’s Hospital of Nanjing Medical University, Huai’an, China

**Keywords:** plasma, circRNA, PD, risk score function, ROC Curve

## Abstract

Parkinson’s disease (PD) is a chronic and progressive degenerative disease of the central nervous system. Degenerative neuropathy can occur in patients with PD even before typical clinical symptoms appear in the preclinical stage. Therefore, if the early diagnosis of degenerative diseases can be timely and the correlation with the disease progression can be explored, the disease progression will be slowed down and the quality of life of patients will be improved. In this study, the circRNA microarray was employed to screen the dysregulated circRNA in plasma samples of PD. Four circRNAs (circ_0085869, circ_0004381, circ_0017204, and circ_0090668) were obtained with increased levels in PD patients by cross comparison and preliminary verification in PD comparing with healthy controls. Further validation found the circRNA panel was consistent with the training set. The ROC curve also revealed a high diagnostic ability of circ_0004381 and circ_0017204 in predicting the early stage of PD from healthy controls. circ_0085869, circ_0004381, circ_0017204, and circ_0090668 also presented a high ability to distinguish the late stage of PD from early stage. In conclusion, circulating circRNA panel might be a potential fingerprint for predicting the early diagnosis of PD and may act as a biomarker for disease progression.

## Introduction

Parkinson disease (PD) is a chronic neurodegenerative disease. The incidence of PD is closely related to population aging (Almaguer-Mederos et al., 2021). With the aggravation of population aging in China, the incidence of PD is also increasing. Currently, the prevalence rate of PD among people over 65 years old in China is about 1.7% (Lin et al., 2021). Motor symptoms of PD mainly include static tremor, bradykinesia, myotonia, and masked face. Non-motor symptoms mainly include hyposmia, constipation, sleep disorders, autonomic nervous dysfunction, and cognitive impairment (He et al., 2021). The accuracy rate of PD early diagnosis is very low; the sensitivity of the clinical diagnosis of PD is about 88% and the specificity is about 68%. The nerve pathology examination is still the gold standard for diagnosis of PD. It is necessary to develop a new diagnosis method of PD or find other related biomarkers for Parkinson’s syndrome (such as multiple system atrophy and progressive paralysis on the nuclear and basal ganglia cortex degeneration) (Lv et al., 2021). Degenerative neuropathy can also occur in patients with PD even before typical clinical symptoms appear in the preclinical stage (Li C. Y. et al., 2021). Therefore, if the degenerative diseases can be diagnosed and treated in a timely manner in the early stage, it will slow down the disease process and improve the quality of life of patients. At present, the diagnostic criteria of PD are mainly based on the clinical symptoms of patients, but the gold standard still needs the confirmation of neuropathology, so it is urgent to find relevant biomarkers for PD diagnosis, disease severity assessment, and prognosis prediction. Blood, cerebrospinal fluid (CSF), and human biological samples for the diagnosis of PD are potential candidates (Li Q. et al., 2021); however, identification of biomarkers in the blood still have some challenges. In addition, the combined application of imaging, metabolomics, proteomics, and gene expression profile can also provide a more meaningful basis for the diagnosis and treatment of PD (Yang et al., 2021).

With the development of high-throughput sequencing technology, researchers have found a class of non-coding RNAs that are stably expressed in human tissues, called circular RNAs (circRNAs) (Zhao et al., 2020). CircRNAs are single-stranded RNA molecules of about 100 nucleotides, which form a circular structure through covalent binding and are stably expressed in eukaryotes (Franz et al., 2019). A large number of circRNAs have been found to be involved in the occurrence and development of human diseases through a variety of regulatory pathways and interact with protein-coding genes in a complex network of mutual regulation. Existing studies have shown that circRNAs can be stable in many kinds of bodies, including blood, saliva, and urine, due to their structural properties (Lin et al., 2019). In addition, circRNAs have been shown to be biomarkers for potential non-invasive diagnoses of atherosclerosis, central nervous system diseases, degenerative diseases, and cancers. However, in PD, only lncRNA and miRNA have been studied as potential diagnostic markers (Huaying et al., 2020); circRNA has not been reported at present. In addition, these characteristics of circRNA provide a theoretical basis for further exploration of potential diagnostic markers in PD.

In this study, we investigated the potential use of circulating cell-free circRNAs in plasma as biomarkers for PD. We not only paid attention to the predictive ability of circRNA in the early diagnosis of PD, but also focused on the predictive ability of circRNA in the progression of PD. According to the Hoehn & Yahr (H&Y) stage classification, we selected healthy patients, H&Y grade 1, H&Y grade 2–3, and H&Y grade 4–5, as grouped objects. After high-throughput detection, the predictive ability of different expressed circRNAs was analyzed for early diagnosis of PD and the predictive ability for progression of PD through multiple validations of the test set and validation set.

## Materials and Methods

### Research Population and Samples

The study enrolled 300 PD patients and 100 healthy individuals in two separate cohorts in The affiliated Huai’an No. 1 People’s Hospital of Nanjing Medical University. All PD patients underwent brain MRI or CT evaluation, excluding related cerebrovascular lesions. According to Hoehn & Yahr classification, patients were divided into 1–5 types. In total, 100 patients with stage 1, 100 patients with stage 2–3, and 100 patients with stage 4–5 were enrolled. The control group was a healthy group without neurological disease or family history of PD identified. Malignant tumors, mental disorders, collagenous diseases, endocrine, cardiovascular disease, or infections were excluded. In addition, patients with atypical Parkinson’s disease were excluded. The diagnosis of PD was based on the latest guideline specimens, and the diagnosis was confirmed by two independent neurologists with identical typing results. This study was approved by an institutional review board of The affiliated Huai’an No. 1 People’s Hospital of Nanjing Medical University. Written informed consent was obtained from all enrolled subjects. All plasma samples were collected using EDTA anticoagulant tubes. Half an hour after sampling, the samples were centrifuged at 3000 rpm for 10 min. The upper plasma was absorbed and stored at −80°C. All patients signed informed consent before sample collection. The relevant clinical data of all patients was summarized in Table 1.

**TABLE 1:** Clinicopathological characteristics of healthy control subjects and patients with Parkinon’s disease.

**Characteristics**	**PD**	**Control**	***P* value**
N	300	100	
Age Mean (SD, year)	65.12(10.31)	66.25 (5.77)	0.21^a^
Gender (male/female)	169/131	51/49	0.35^b^
Diabetes (yes/no)	82/218	29/71	0.75^b^
Hypertension (yes/no)	101/199	42/58	0.13^b^
Age of onset (SD, years)	60.33 (8.12)		
Disease duration (SD, years)	4.79 (2.16)		
Unified Parkinson’s Disease Rating Scale part III	26.12 (1.78)		
Mini-Mental State Examination (SD)	27.12 (1.89)		
Hoehn & Yahr (SD)	2.38 (0.35)		
Levodopa equivalent daily dose (SD, mg)	499.69 (60.01)		

*^*a*^Student *t*-test. ^*b*^Chi-square test.*

### CircRNA Microarray and Analysis

RNA was extracted from three plasma samples diagnosed with PD and three healthy controls as circulating samples, both of which were used for microarray detection. Each sample was tested with a 1.0 μg total RNA. The microarray was detected by using Human CircRNA Microarray V2 (CapitalBio, Beijing, China). We screened out all the low-expression genes before performing other analyses. We only retained genes with at least half markers in five out of samples. This reduces the initial 37,681 input genes in each sample to about 15,000 detected genes. A bilateral Mann-Whitney *U* test performed by Wilcox was employed. Finally, we use the Benjamin-Hochberg correction (R. P. Adojust function) to explain the multiple tests. For the purpose of analysis, we found that the genes dysregulated were those with a Benjamin-Hochberg Q value < 0.05. The Limma package was further used for parallel differential expression analysis. All data analysis and presentation was performed using custom R scripts.

### RNA Extraction and Quantitative Real-Time PCR (qRT-PCR)

We used Trizol (Invitrogen, CA, United States) method for RNA extraction, and the specific method was carried out according to manufacturer’s instructions. After RNA extraction, its concentration and purity were determined by ND-1000 (Thermo, CA, United States), and RNA was clarified by 1% agarose electrophoresis to ensure RNA integrity. In order to clarify the stability of circRNA expression, we used the combination of internal and external parameters for correction. The internal reference was GAPDH and the external reference was cel-miR-39 (ABI, CA, United States). The circRNA was amplified by random primer method and amplified in ABI 7900 (ABI, CA, United States). The relative expression of circRNA was calculated using the 2^–ΔΔCq^ method.

### Screening Phase

The screening phase was divided into a training set and validation set. In each group, 20 samples were collected while the validation set contained another 80 samples in each group. Groups were named as healthy volunteers and PD patients.

Risk score analysis was used to analyze the predictive power of certain circRNAs. In brief, the upper 95% reference interval (95% CI) of each circRNA value in the control group was used as the cut-off value for a certain circRNA expression level. If circRNA expression was higher than 95% CI in this sample, we rated it as 1, and if it was lower than 95% CI, we rated it as 0. The risk score was defined as a linear combination of the expression levels of each circRNA. The risk score for the four circRNAs was calculated using weight regression coefficients derived from univariate logistic regression analyses for each circRNAs. The samples were ranked by RSF and then divided into a high-risk group (representing the PD group) and a low-risk group (representing the predictive control individuals).

### Statistical Analysis

All statistical analyses were performed using SPSS 13.0 software (Chicago, IL, United States) and GraphPad Prism software (La Jolla, CA, United States). The chi-square test was used to analyze the correlation of clinical features of the patients. The differences between groups were analyzed using an unpaired *t*-test when only two groups were compared and a one-way ANOVA analysis of variance when more than two groups were compared. Differences were considered to be statistically significant at *P* < 0.05.

## Results

### The circRNA Expression Landscape in PD and Healthy Control

**FIGURE 1:**
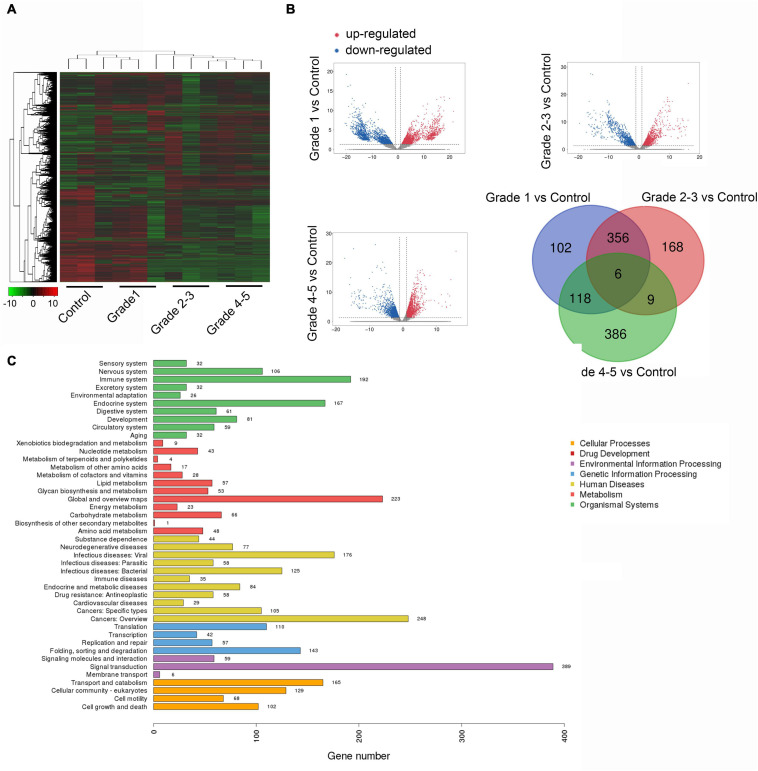
The landscape of circRNA expression in plasma sample of PD and healthy controls. **(A)** Cluster analysis of differently expressed circRNAs (three plasma samples from patients diagnosed with PD and three healthy controls). **(B)** The scatter plots of dysregulated circRNAs in different stage of PD comparing with healthy control. **(C)** Candidate gene pathway enrichment.

After microarray detection, we obtained a remarkably differently expressed circRNA landscape in the plasma samples of PD patients and healthy controls. We divided the PD patients into three groups according to the Hoehn & Yahr stage. Stage 0 was excluded because of no obvious clinical symptoms. Stage 1 was defined as early stage while stage 2–3 and 4–5 were defined as late stage. As presented in Figure 1A, hierarchical clustering analysis revealed a different expression profile of circRNA in PD with different stages. We next compared the PD with different stages with healthy controls. As presented in Figure 1B, we used the following parameters for further screening: *P* value < 0.05; CT value < 35; and detection rate > 75%. A total of 582 circRNA transcripts were specifically increased in the PD stage 1 patients group compared with control group, 539 circRNAs were proven to be upregulated in stage 2–3 patients, while 519 circRNA were evaluated in stage 4–5 patients. In order to reveal the potential biomarkers for PD early diagnosis and monitor disease progression, we selected the circRNA with upregulation in all the PD patients comparing with healthy controls. The Venny method was applied to obtain six candidate circRNAs. We compared the dysregulated circRNA in PD patients with different stages with healthy controls. After we obtained the upregulated circRNA in PD patients with different stages, we applied the Venny method to screen the circRNAs that increased in all three groups. After analyzing, we obtained six circRNA, namely circ_0085869, circ_0055327, circ_0046064, circ_0004381, circ_0017204, and circ_0090668, as candidates for further investigation. The pathway analysis through ceRNA method was applied; the top three pathways enriched were the sensory system, nervous system, and immune system (Figure 1C).

### Differently Expressed circRNA in PD Samples

**FIGURE 2:**
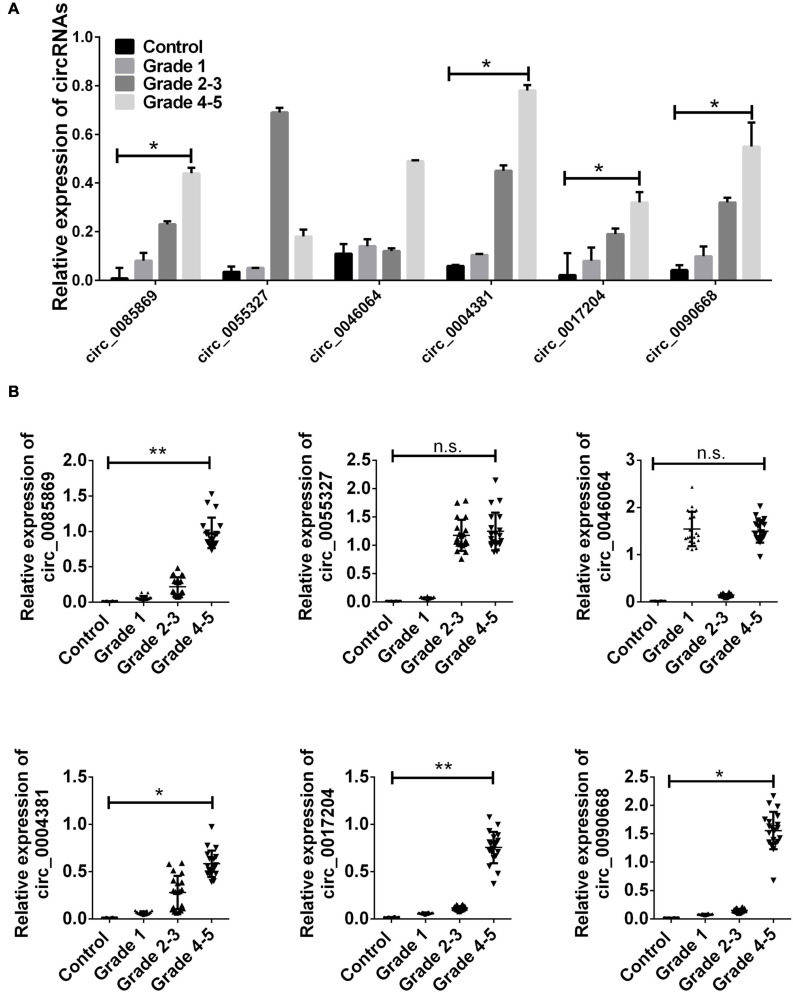
CircRNA expression in training set. **(A)** The relative expression level of six circRNAs in PD patients and healthy control used in microarray detection. **(B)** Total 20 paired plasma from PD patients in each group, and 20 controls were used in RT-qPCR analysis. Data was presented as mean ± SEM. Data was analyzed with student *t*-test. n.s. indicated no significant, * indicated *p* < 0.05 and ** indicated *p* < 0.01.

After we obtained the six circRNAs as candidates, we firstly examined the expression of these circRNAs in the samples enrolled during microarray detection. We found that only circ_0085869, circ_0004381, circ_0017204, and circ_0090668 presented rapidly increased levels along with the disease progression (from stage 1 to stage 4–5). No difference was obtained for circ_0055327 between stage 2–3 and stage 4–5 while for circ_0046064 no difference was found between stage 1 and stage 2–3 (Figure 2A).

Next, we employed the two staged validation including the training set and validation set to further examine the different expressions of the four circRNA panel in PD patients and healthy controls. Initially, 20 randomly selected samples in each group was labeled as the training set. The upregulation of the four circRNA panel was confirmed in PD patients with stage 1 comparing with healthy controls. In addition, we also obtained an evaluated level of these circRNAs in different stages of PD patients (Figure 2B).

**FIGURE 3:**
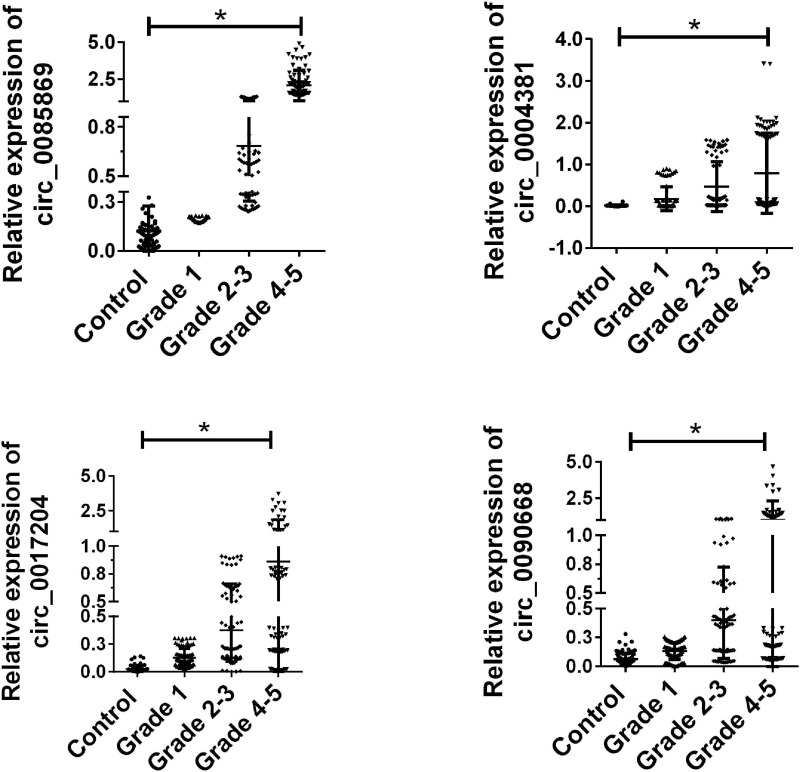
Validation of candidate circRNA in validation set. Total 80 paired plasma from PD patients with different stages, and 80 healthy controls were used in RT-qPCR analysis. Data was presented as mean ± SEM. Data was analyzed with student *t*-test, * indicated *p* < 0.05.

Based on the results in the training set, the remaining 80 paired samples were enrolled as a validation set. We next examined the expression of the four candidate circRNAs in the validation group. As presented in Figure 3, circ_0085869, circ_0004381, circ_0017204, and circ_0090668 were confirmed with higher expression levels in PD and were increased when the stage developed.

**FIGURE 4:**
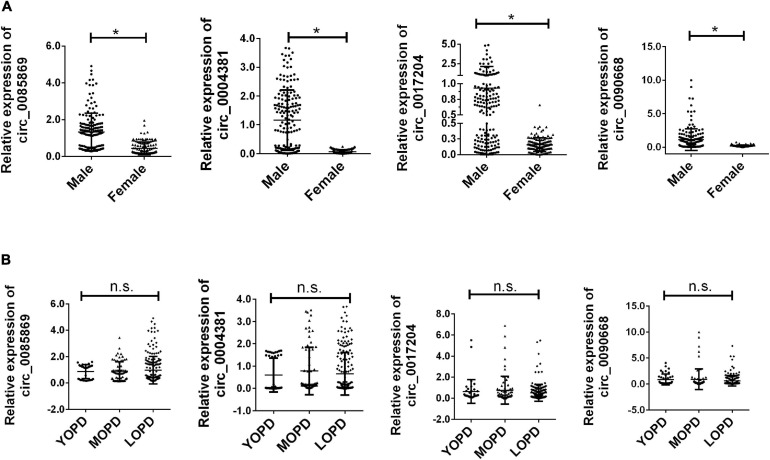
Relative expression of circRNA panel in PD subgroups. **(A)** Relative expression of circRNA panel in males and females of PD. **(B)** Relative expression of circRNA panel in PD for different age groups of onset. Data was presented as mean ± SEM. * indicated *p* < 0.05, n.s. indicated no significant.

Previous studies have shown that the incidence of PD has an obvious gender bias. We further divided the PD patients into male and female subgroups. The further analysis of the expression of the four circRNA with different stage in males and females has also been applied. We found that there was a significant increased level of circRNAs in males compared with females. All the PD patients were also divided into three groups: young onset (YOPD, ≤ 49 years old), middle onset (MOPD, 50–60 years old) and late onset (LOPD, > 60 years old). However, the four circRNAs presented no significant difference for different age groups in each dataset (Figure 4).

### Diagnostic Capability Assessment

**FIGURE 5:**
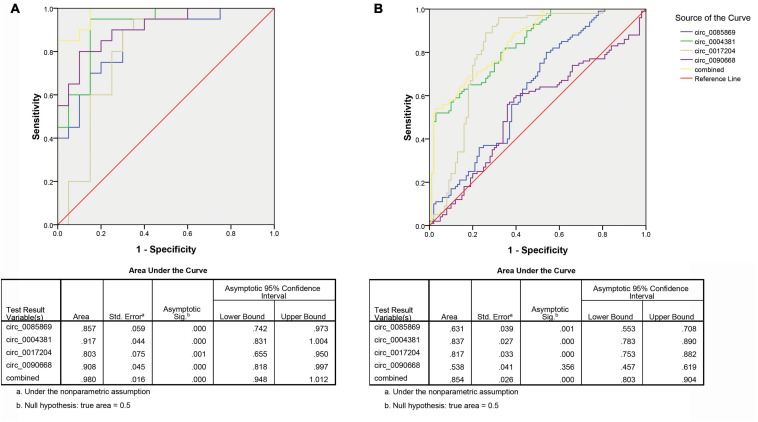
ROC analysis of circRNA panel predicting early PD (stage 1) by using risk score analysis. **(A)** Training set. **(B)** Validation set.

In order to further explore the accuracy and specificity of these three circRNAs as potential signatures, risk scoring formulas were used to evaluate the diagnostic value of the four circRNAs. Firstly, we analyzed the prediction ability for early diagnosis of PD. We compared the expression level of circRNAs in PD with stage 1 and healthy controls. We divided the control and case groups in the training set according to the upper 95% confidence interval (95% CI) of the control group. Logistic regression analysis was used to calculate the risk score. All plasma samples were then divided into a high-risk group (possibly PD patients with stage 1) and a low-risk group (predicted to be a control group). We defined the cutoff value as the maximum of sensitivity + specificity. The positive predictive value (PPV) and negative predictive value (NPV) calculated in the training set were 90% and 85%, respectively. We further applied the same values to calculate the risk score for the validation set sample, with PPV and NPV of 86% and 85%, respectively (Table 2). In addition, we also used ROC curve analysis to evaluate the predictive diagnostic value of circRNA for PD (stage 1). In the test set, the areas under the ROC curve of the circRNAs as well as the combination of the four circRNA were 0.857, 0.917, 0.803, 0.908, and 0.980, respectively (Figure 5A). In the validation set, the areas under the ROC curve of the circRNAs as well as the combination of the four circRNA were 0.631, 0.837, 0.817, 0.538, and 0.854, respectively, indicating that circ_0004381 and circ_0017204 presented a good ability to distinguish the early stage of PD (stage 1) patients from the control group (Figure 5B).

**TABLE 2:** Risk score analysis of in PD and cancer-free control plasma samples.

**Score**	**0–6.89**	**6.89–13.71**	**PPV^a^**	**NPV^b^**
**Training set** (***n* = 20)**			0.90	0.85
PD (stage 1)	2	18		
Control	17	3		
**Validation set (*n* = 80)**			0.86	0.85
PD (stage 1)	11	68		
Control	69	12		

*^*a*^PPV, positive predictive value. ^*b*^NPV, negative predictive value.*

**FIGURE 6:**
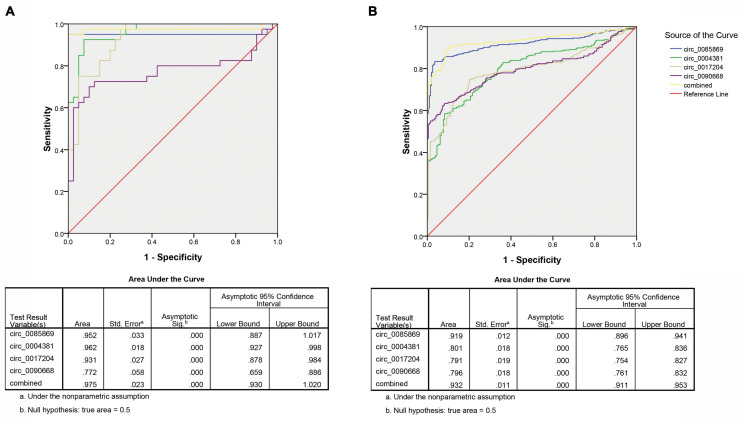
ROC analysis of circRNA panel predicting late PD (stage 2–5) by using risk score analysis. **(A)** Training set. **(B)** Validation set.

Secondly, we measured the diagnosis ability for the four circRNA in predicting the development of PD. We pooled the PD patients with stage 2–3 with stage 4–5 as late stage of PD and compared them with the early stage (PD stage 1). The same risk score analysis was also applied. The PPV and NPV calculated in the training set were 95% and 97%, respectively. We further applied the same values to calculate the risk score for the validation set sample, with PPV and NPV of 81% and 85%, respectively (Table 3). The AUC for the circRNA panel as well as the combination of the four circRNA in training set were 0.952, 0.962, 0.931, 0.772, and 0.975, respectively (Figure 6A). In the validation set, the AUC were 0.919, 0.801, 0.791, 0.796, and 0.932, respectively, indicating that circ_0085869, circ_0004381, circ_0017204, and circ_0090668 had a good effect of distinguishing late PD (stage 2–5) patients from the early PD (Figure 6B).

**TABLE 3:** Risk score analysis of in PD with different stages.

**Score**	**0–4.55**	**4.55–8.91**	**PPV^*a*^**	**NPV^*b*^**
**Training set (*n* = 60)**			0.95	0.97
PD (stage 2–5)	3	58		
PD (stage 1)	57	2		
**Validation set (*n* = 240)**			0.81	0.85
PD (stage 2–5)	45	205		
PD (stage 1)	195	35		

*^*a*^PPV, positive predictive value. ^*b*^NPV, negative predictive value.*

### Endogenous Expression Stability Investigation

**FIGURE 7:**
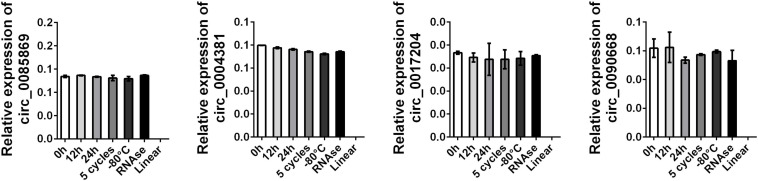
Stability detection of circRNA panel in human plasma. RT-qPCR was applied for detecting the expression level of the four circRNAs. Data was presented as mean ± SEM with log-transformed. No significant difference was observed in each group.

Next, we selected three healthy control plasma samples for stable expression detection. The above-mentioned plasma samples were subjected to the following different treatments: storage at room temperature for 0 h, 12 h, and 24 h; repeated freeze-thaw cycles for 5 days; storage at −80°C for 7 days; and digestion with RNase. RNA was then extracted from the samples for amplification. We also detected the linear style of the four circRNAs by using the same primers. As presented in Figure 7, we found the endogenous expression levels of the four circRNAs remained unchanged while the linear RNA was not amplified.

## Discussion

PD, also known as palsy tremor, is one of the most common neurodegenerative diseases in middle-aged and elderly people (Bailey et al., 2021; Lin et al., 2021). The prevalence increases significantly with age. The main pathological changes are the degeneration and loss of dopaminergic neurons in the substantia nigra and the formation of lewy bodies (Lv et al., 2021). Clinical manifestations can be divided into motor symptoms and non-motor symptoms. Motor symptoms include static tremor, myotonia, bradykinesia, and balance disorders. Non-motor symptoms include autonomic nervous dysfunction, sleep disorders, and depression (Das et al., 2021). Currently, there is still a lack of clear indicators for early diagnosis of PD, and the specificity for early diagnosis of PD is not high according to clinical symptoms. Combining cerebrospinal fluid biochemical markers, blood biochemical markers, and functional neuroimaging, can improve the accuracy of early diagnosis of PD.

A circulating biomarker for the early diagnosis or the dynamic monitoring of PD has been identified recently. phosphorylated α-syn can be detected in blood plasma and shows more promise as a diagnostic marker than the non-phosphorylated protein (Foulds et al., 2011). Studies have also shown that the level of GSH in the substantia nigra decreases before clinical symptoms appear in PD patients (Wei et al., 2020). Therefore, GSH can be used as a marker for the early diagnosis of PD. Cristalli et al. measured the erythrocyte GSH peroxidase content in 211 PD patients and 135 healthy people and found that the content in PD patients decreased significantly compared with the control group and was positively correlated with the progression of the disease (Cristalli et al., 2012). For circulating non-coding RNA acting as biomarkers for PD, miRNA, and lncRNA have been reported to hold a certain ability, especially for early diagnosis of PD. Researchers have proven a subset of miRNAs including upregulation of miR-27a and downregulation of let-7a, let-7f, miR-142-3p, and miR-222 which may aid in the early diagnosis of PD (Chen et al., 2018; Quan et al., 2020). The decreased level of lncRNA MEG3 was also obtained in the plasma of PD patients and was proven to be highly associated with PD stage. The H&Y scale was also applied to define the PD stage (Quan et al., 2020). The current study shows that the occurrence of PD is different in gender, and it is also correlated with the onset age to a certain extent. Previous studies have shown that miRNAs, which can be used as plasma markers, are also gender-dependent in the expression of PD (Piscopo et al., 2021). The biological significance of this sex association has not been clarified, but an increase in the prevalence of PD in men has been recognized. Bai et al. reported that serum miR-29a and miR-29c tended to decrease with the severity of PD (Bai et al., 2017). In our study, it was also found that circRNA was abnormal and highly expressed in male PD patients, which was consistent with previous relevant reports to some extent, but there was no relevant difference in circRNA at different age of onset. The reasons for this need to be further verified by subsequent studies. For circRNA in plasma samples of PD, little was known on whether it could be used as biomarker. However, the expression of circRNA peripheral blood mononuclear cells of patients diagnosed with PD has been investigated. Among the six aberrantly expressed circRNA, four could distinguish PD from healthy control (Ravanidis et al., 2021).

Current research on biological markers of PD focuses on neuroimaging combined with biochemical markers (Sinclair et al., 2021). Although a perfect biomarker has not yet been found, great progress has been made in the search for biomolecules and image types. At present, DAT is the most widely used imaging method for the diagnosis of PD, but the ability of differential diagnosis needs to be improved (Bannister et al., 2021). The advantage of biochemical markers is that they can fill in the gaps in the field of imaging and reveal new biological targets for the monitoring of PD disease progression (Yu et al., 2021). The availability of biochemical markers in body fluids is an advantage, making it a routine clinical test. Future efforts should be directed toward a combination of these two fields. And research needs to extend to the early symptoms of PD. Combining imaging and biochemistry can improve the accuracy of clinical diagnosis, including prodromal symptoms, and can monitor disease progression and the effectiveness of treatment, which makes more sense in the early stages when neuroprotective therapy is most effective.

Due to the limitation of the sample size, this study is only a relatively preliminary conclusion at present. Future studies will expand the sample size for verification and further explore the specific functional mechanism of candidate circRNAs.

## Conclusion

In summary, we used a microarray-based approach to screen the potential fingerprints of PD. We found that the circ_0004381 and circ_0017204 panel may be able to predict early stage of PD in the normal population with relatively high sensitivity and specificity, while circ_0085869, circ_0004381, circ_0017204, and circ_0090668 could distinguish late PD from early PD acting as a dynamic monitoring factor for the development of PD. However, due to the limitation of the sample size in this study, more samples are needed for verification, and further studies are needed to confirm the potential regulatory mechanism of these circRNAs in the development of PD.

## Data Availability Statement

The datasets presented in this study can be found in online repositories. The names of the repository/repositories and accession number(s) can be found in the article/supplementary material.

## Ethics Statement

The studies involving human participants were reviewed and approved by the Institutional review board of The affiliated Huai’an No. 1 People’s Hospital of Nanjing Medical University. The patients/participants provided their written informed consent to participate in this study. Written informed consent was obtained from the individual(s) for the publication of any potentially identifiable images or data included in this article.

## Author Contributions

LZ and KJ: manuscript writing, literature search, and data analysis. AC and HC: data analysis and statistical analysis. LZ: research design. All authors read and approved the final manuscript.

## Conflict of Interest

The authors declare that the research was conducted in the absence of any commercial or financial relationships that could be construed as a potential conflict of interest.

## Abbreviations

PD: Parkinson’s disease
H&Y stage: Hoehn& Yahr stage
circRNA: circular RNA
qRT-PCR: quantitative real-time polymerase chain reaction
ROC curve: Receiver Operating Characteristic curve
AUC: Area under the Curve
RSF: Risk score function.
